# Isolation of a Novel Thermophilic Methanogen and the Evolutionary History of the Class *Methanobacteria*

**DOI:** 10.3390/biology11101514

**Published:** 2022-10-16

**Authors:** Zhenbo Lv, Jiaxin Ding, Heng Wang, Jiaxin Wan, Yifan Chen, Lewen Liang, Tiantian Yu, Yinzhao Wang, Fengping Wang

**Affiliations:** 1 State Key Laboratory of Microbial Metabolism, School of Life Sciences and Biotechnology, Shanghai Jiao Tong University, Shanghai 200240, China; 2 Instrumental Analysis Center, Shanghai Jiao Tong University, Shanghai 200240, China; 3 School of Oceanography, Shanghai Jiao Tong University, Shanghai 200240, China; 4 Southern Marine Science and Engineering Guangdong Laboratory (Zhuhai), Zhuhai 519000, China

**Keywords:** methanogenic archaea, enrichment and isolation, comparative genomic, metabolic pathway reconstruction, thermal adaptation, evolutionary history

## Abstract

**Simple Summary:**

The methanogenesis pathway via methanogens dates back to the Hadean or Archaean Earth. These methanogens are considered to have a thermophilic origin and are presently ubiquitously distributed across anaerobic environments. The class *Methanobacteria* comprises methanogens that are found extensively in geothermal environments, such as hot springs and hydrothermal vents, and their evolutionary history and how they adapted to different temperatures remain unclear. In this study, we isolated a novel species of the class *Methanobacteria* from a natural hot spring in Tengchong, China. This species can produce methane, utilizing hydrogen and carbon dioxide, at 65 °C. In addition, we found that members of the class *Methanobacteria* originated in a geothermal niche and then evolved to adapt to ambient temperatures; during this process, thermal adaptation genes were lost and a wide range of metabolic genes were acquired. This research on methanogen evolution will help us understand how life originated in geothermal environments and then spread extensively across present-day Earth.

**Abstract:**

Methanogens can produce methane in anaerobic environments via the methanogenesis pathway, and are regarded as one of the most ancient life forms on Earth. They are ubiquitously distributed across distinct ecosystems and are considered to have a thermophilic origin. In this study, we isolated, pure cultured, and completely sequenced a single methanogen strain DL9LZB001, from a hot spring at Tengchong in Southwest China. DL9LZB001 is a thermophilic and hydrogenotrophic methanogen with an optimum growth temperature of 65 °C. It is a putative novel species, which has been named *Methanothermobacter tengchongensis*—a Class I methanogen belonging to the class *Methanobacteria*. Comparative genomic and ancestral analyses indicate that the class *Methanobacteria* originated in a hyperthermal environment and then evolved to adapt to ambient temperatures. This study extends the understanding of methanogens living in geothermal niches, as well as the origin and evolutionary history of these organisms in ecosystems with different temperatures.

## 1. Introduction

Methanogens are considered one of the most ancient life forms on Earth, with an evolutionary history dating back more than 3.4 billion years [1,2]. The methanogenesis pathway is considered one of the most ancient metabolic pathways and may even be shared by the last universal common ancestor (LUCA) [3]. The methanogenesis pathway can be classified into four major types, based on metabolic substrate utilization, i.e., hydrogenotrophic (H_2_/CO_2_ and formate), methylotrophic (methanol, methylamine, methanethiol), acetoclastic (acetate), and other complex carbon sources such as coal [4,5]. It has been estimated that about one billion tons of methane are produced via these methanogenesis pathways and enter the atmosphere every year, accounting for ~1.6% of annual global carbon fixation [6]. Methane is the second most potent greenhouse gas on Earth and significantly influences global climate change [7]. Therefore, methanogens are crucial for the stability of Earth’s ecosystems [6], and increasing attention has recently been focused on methanogens.

Previous studies have shown that the methanogenesis pathway may have existed in the common ancestor of the Euryarchaeota and TACK superphyla, including Ca. Verstraetearchaeota [8], Ca. Bathyarchaeota [9], Ca. Nezhaarchaeota and Thaumarchaeota [10,11], close to the last common archaea ancestor [12], which has been postulated to be a thermophilic methanogen [8,9,10,11,13]. However, none of the TACK methanogens have been pure cultured, and their actual optimum growth temperatures have not been verified. Pure cultured methanogen strains to date have only been from the phylum Euryarchaeota, and can be categorized into three major groups: (1) the Class I methanogens, which include three classes (*Methanobacteria*, *Methanococci,* and *Methanopyri*); (2) Class II methanogens, referred to as the class *Methanomicrobia*; (3) Class III methanogens, including the order *Methanomassiliicoccales*, Ca. Methanofastidiosales and Ca. Nuwarchaeales [12,14,15]. Most Class II and III methanogens are found in temperate environments, while many of the Class I methanogens are thermophilic and can be found in hydrothermal vents or hot springs [12,14,15]. Within the Euryarchaeota group, the class *Methanobacteria*—a Class I methanogen—comprises thermophilic and non-thermophilic members [16,17,18,19,20]; however, the evolutionary history of this ancient methanogen branch remains elusive.

The isolated strains in the class *Methanobacteria* have diverse temperature adaptation ranges and are divided into five genera: *Methanothermus*, *Methanothermobacter, Methanobrevibacter*, *Methanosphaera,* and *Methanobacterium* [16,17,18,19,20]. The deep-branching genus *Methanothermus* can grow on H_2_ and CO_2_, and its optimum growth temperature range is 80–88 °C [20,21]. In contrast, the optimum growth temperature for *Methanothermobacter* is 55–70 °C, with H_2_ or formate and CO_2_ as metabolic substrates [17,22]. However, the genera *Methanobrevibacter*, *Methanosphaera,* and *Methanobacterium* have optimum growth temperature ranges of 28–40 °C [18,23,24,25]. It remains unclear whether the class *Methanobacteria* has a thermophilic origin or how their descendants adapted to temperate environments. In this study, we isolated and pure cultured a single *Methanobacteria* strain DL9LZB001, of the genus *Methanothermobacter,* from a hot spring in Tengchong, China. DL9LZB001 is postulated as a novel species and has been named *Methanothermobacter tengchongensis*. We sequenced and analyzed its genome to deepen our understanding of the evolutionary history of the class *Methanobacteria*.

## 2. Materials and Methods

### 2.1. Sampling, Enrichment, Isolation, and Pure Culturing

Our samples were collected from a hot spring in Tengchong, China (98.82350° N, 25.04233° W) in January 2020. The temperature of the sampling site was 65.7 °C, and the pH was 6.66. Sediments and upper water were collected and stored in anaerobic bottles with nitrogen as the headspace.

During the primary enrichment, we mixed each set of sediments collected with its corresponding upper water sample in an anaerobic tank. We transferred 100 mL of the mixture into 125 mL sterile anaerobic bottles with butyl rubber stoppers and then amended it with various substrates, such as methylamine, sodium formate, and methanol. We pressurized H_2_ (100%, 10 psi) into these bottles and maintained them at 65 °C for approximately one year. Then, we inoculated the primary enriched product into a modified MB medium [26], and each group was enriched with the corresponding previously amended substrates. The modified MB medium contained the following, per liter: 900 mL distilled water, 1.00 g NH_4_Cl, 0.30 g K_2_HPO_4_·3H_2_O, 0.30 g KH_2_PO_4_, 0.20 g KCl, 0.50 g MgCl_2_·6H_2_O, 2.00 g NaCl, 0.05 g CaCl_2_, 1.00 mL resazurin solution (0.5 g·L^−1^), and 1.00 mL trace element solution [26,27]. After the medium was autoclaved and then cooled, we transferred a 100 mL mixture solution (containing 1.00 mL vitamin mixture solution [27], 4.00 g bicarbonate, and 0.5 g Na_2_S·9H_2_O) into the medium via a 0.22 μm filter in the anaerobic tank. The pH value was regulated to ~7. We then inoculated approximately 5 mL of enriched product into a 95 mL modified MB medium in 125 mL bottles in an anaerobic tank, maintained at 65 °C for 30 days before the next round of enrichment.

The strains were isolated using a Hungate rolling tube [28] by adding 2% gellan gum (*w*/*v*), 1.00 g·L^−1^ yeast extract, 2.00 g·L^−1^ tryptone, 10 mM methylamine, and 10 mM sodium formate into the modified MB medium described earlier; H_2_ (100%, 10 psi) was then pressurized into the tube. Subsequently, all the colonies—with different colors, sizes, and shapes—from the middle of the tube were collected and transferred into a 5 mL modified MB medium with additional methylamine, sodium formate, yeast extract, and tryptone, in 20 mL bottles filled with H_2_/CO_2_ (80%/20%, 10 psi). We repeated this process three times and used antibiotics for purification, including ampicillin sodium, kanamycin sulfate, streptomycin sulfate, and vancomycin hydrochloride at the same final concentration of 200 mg·L^−1^. Finally, we serially cultured the strains in a modified MB medium with H_2_, CO_2_, and bicarbonate as the only energy and carbon sources. The cell concentrations were monitored spectrophotometrically at an optical density of 600 nm (OD_600_), with 1 mL double distilled water as the blank. The pH range for growth in the modified MB medium was determined to be 4.0–10.0, by using different pH buffers: (1) 0.1 M citrate and 0.1 M sodium citrate for pH 4 and pH 5; (2) 0.2 M Na_2_HPO_4_ and 0.2 M NaH_2_PO_4_ for pH 6, pH 7, and pH 8; (3) 0.1 M NaHCO_3_ and 0.1 M Na_2_CO_3_ for pH 9 and pH 10 [29]. The salinity range for cell growth was determined at NaCl concentrations of 2, 10, 20, 30, and 40 g·L^−1^, at 0.2%, 1%, 2%, 3%, and 4% (*w*/*v*), respectively.

### 2.2. Headspace Gas Analysis

The methanogenesis of the enrichments and cultures was monitored using a gas chromatograph (GC, Agilent, California, USA) and a flame ionization detector (FID). The injection temperature, column temperature, and FID temperature were 60 °C, 80 °C, and 300 °C, respectively. Before monitoring the samples, we tested pure methane (99.9%) at concentrations of 0%, 20%, 40%, 60%, 80%, and 100% to establish a standard curve between various concentrations of methane and the peak areas of the GC results. Subsequently, the methane concentrations in the headspace of the samples were calculated using the results for the corresponding peak area.

### 2.3. Microscopy Observation

The coenzyme F420 contains a fluorescent component [30], and the oxidation states of the coenzyme F420 can absorb 420 nm ultraviolet light and excite 470 nm blue-green fluorescence [30]. Isolated cells from pure cultures were observed under a fluorescence microscope (BX63 Olympus, Tokyo, Japan) with 405 nm ultraviolet light as the light source. For observation under electron microscopes, we centrifuged the cell suspension at 6000× *g* for one minute and washed it three times with 0.5 mL sterile water. We then fixed the cells on a silicon pellet illuminated by a white light source and observed them under a scanning electron microscope (VEGA 3, TESCAN, Brno-Kohoutovice, Czech Republic). Alternatively, the cells were fixed on copper grids and observed under a transmission electron microscope (Tecnai G2, Thermo Fisher Scientific, Waltham, MA, USA).

### 2.4. ToF-SIMS Analysis

Cell morphology and composition were imaged using time-of-flight secondary ion mass spectrometry (ToF-SIMS) analysis. ToF-SIMS can directly distinguish between different cell-surface components because of its high mass resolution and spatial resolution. For this study, we used ION-ToF ToF-SIMS 5 at the Shanghai Jiao Tong University Instrument Analysis Centre, and the pressure of the analysis chamber was maintained below 1.1 × 10^−^^9^ mbar. The fast imaging mode with a pulsed 30 keV Bi^3+^ (0.16~0.18 pA pulsed current) ion beam was applied for high lateral resolution mapping (<100 nm) analysis, which showed the 2D or 3D distribution of elements and molecules in cells; the typical analysis area was 50 × 50 μm^2^, the lateral resolution as low as 60 nm, and the sensitivity as high as ppm–ppb. Through spectrometry analysis, the specific peaks of target cells, i.e., C_2_SO_2_^+^, PO_3_^−^, and PO_2_^−^, were identified. The distribution of C_2_SO_2_^+^, PO_3_^−^, and PO_2_^−^ acquired by the SIMS mapping method only instructed the distribution of target cells, and even some non-target cells existing in samples, as cells’ chemical composition could be differentiated at the same position. Therefore, ToF-SIMS analysis can be seen as a combination between electron microscopy and mass spectrum analysis, and is presented as a supplementary certification of the TEM/SEM results.

### 2.5. DNA Extraction, Sequencing, and Analysis

DNA was extracted from the pure culture using a Bacterial Genome DNA Rapid Extraction Kit (Huiling Biotechnology Co., Ltd., Shanghai, China), following the manufacturer’s instructions. The exact concentration of the DNA was measured using a Qubit 4 Fluorometer (Thermo Fisher Scientific, Waltham, MA, USA), and DNA purity and integrity were detected via agarose gel electrophoresis. Genome sequencing was completed using PacBio sequencing technology and Illumina PE150 sequencing for high-accuracy assembly. The genome was assembled using SMRT Link software (version 5.0.1) [31], and the protein-coding genes were predicted using GeneMarkS software (Version 4.17) [32]. The tRNA was predicted by the software tRNAscan-SE (version 1.3.1) [33], the rRNA genes were predicted using RNAmmer software (version 1.2) [34], and small RNA was identified against the Rfam database [35] using CMsearch (version 1.1rc4) [36]. The genomic island was predicted using IslandPath-DIOMB (version 0.2) [37], and the CRISPR (clustered regularly interspaced short palindromic repeat) sequences were predicted using CRISPRdigger software (version 1.0) [38]. A display of the entire genomic map was generated using Circos (version 0.69) [39], along with the prediction results of the protein-coding genes.

### 2.6. Phylogenetic Analyses

The genome of our isolated strain and 233 reference genomes of representative archaea from the four superphyla, i.e., Euryarchaeota, TACK, Asgard, and DPANN, were employed to construct a phylogenomic tree based on the concatenated alignment of 37 marker genes [10,40]. The 233 reference genomes were downloaded from the NCBI prokaryote genome database [10]. Each of the 37 marker protein sequences translated from reference genomes was aligned using the rapid multiple sequence alignment program MAFFT (version 7.313), filtered using trimAI software (version 1.4. rev2) [10,41,42], and then concatenated into a single alignment. The phylogenetic tree was then constructed using IQ-Tree software (version 1.6.6) [43], with the model LG + C60 + F + G and a bootstrap value of 1000 [10]. We also downloaded from the NCBI database the completely sequenced genomes of all the isolated strains from the classes *Thermococci*, *Methanobacteria*, *Methanococci,* and *Methanopyri*, and collected in situ environmental and pure culture information on these genomes. A phylogenetic tree for evolutionary analysis was constructed using only the completely sequenced genomes of Class I methanogens, employing the same method.

### 2.7. Comparative Genomic and Ancestral Analyses

The amino acid identities (AAI) of the isolated strains of the classes *Thermococci*, *Methanobacteria*, *Methanococci*, and *Methanopyri* were calculated using the CompareM (version 0.0.23, https://github.com/dparks1134/CompareM (accessed on 12 May 2022)) software toolkit, and the average nucleotide identities (ANI) were calculated using fastANI software (version 1.33) [44]. The protein sequences of all the completely sequenced genomes were predicted using Prodigal gene prediction software (version 2.6.3) [45]. We then identified all the clusters of orthologous groups (COG) of these strains using Orthofinder software (version 2.5.4) [46], and annotated these COG using BlastKOALA (https://www.kegg.jp/blastkoala/ (accessed on 24 June 2022)). Combined with the phylogenetic tree of the isolated strains, the gene sets of the ancestor nodes were predicted using a posterior probability algorithm in Count software (version 9.1106 RC1) [47]. Finally, by comparing the gene sets of different ancestor nodes, we identified the lost and the gained gene sets of each predicted ancestor node.

## 3. Results and Discussion

### 3.1. Enrichment and Isolation of a Thermophilic Methanogen from a Hot Spring

**Figure 1:**
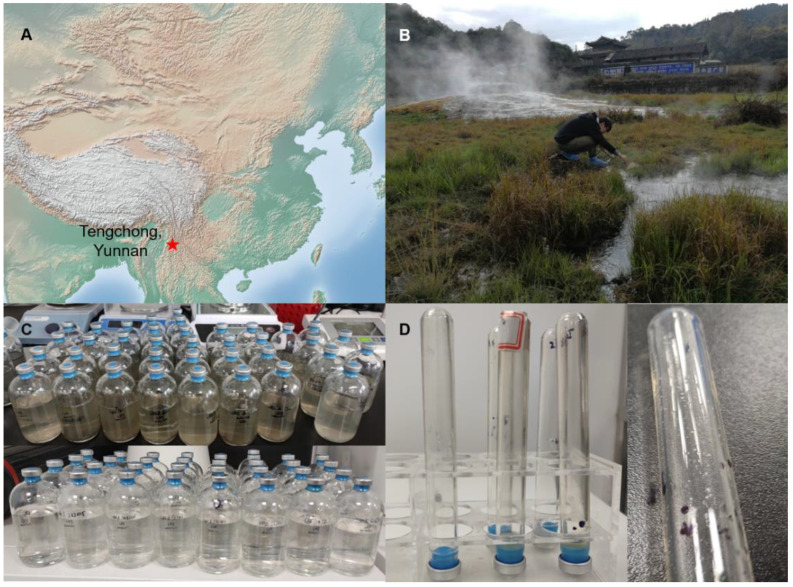
Sampling, enrichment, and isolation of methanogens from hot spring sediments. (**A**) Tengchong is located in the Yunnan province in Southwest China. (**B**) The sampling site was a natural hot spring and the sediments were brownish. (**C**,**D**) We enriched methanogens with methanogenesis substrates, and isolated methanogens using Hungate rolling tubes.

The sediment and upper water samples were collected from a hot spring in Tengchong, China in January 2020 (Figure 1A). This hot spring (pH: 6.66, temperature: 65.7 °C) is a natural environment with abundant vegetation (Figure 1B). Initial enrichment was made using the collected sediment and corresponding upper water sample combined with potential methanogenesis substrates (for details, see Section 2.1) for approximately one year (Figure 1C). A modified MB medium was used in the subsequent enrichments to gradually remove the sediments from the medium (Figure 1C) [26]. To eliminate bacteria from the enrichments, cultures with H_2_/CO_2_ as the headspace gas and bicarbonate as the only carbon source in a liquid medium were established using four different kinds of antibiotics, and rapid methane production was detected. After this round of enrichment, the Hungate rolling tube technique was performed, and one strain of methanogens from this enrichment was successfully isolated; the colony was white, approximately 1–2 mm in diameter, and convex (Figure 1D). After another two rounds of colony isolation via the Hungate rolling tube, and several instances of continuous culture, the strain was purified by checking the cell morphology under an optical microscope and an electron microscope. During continuous culture, the only carbon sources in the medium were CO_2_ and bicarbonate, without any organics, indicating that the strain DL9LZB001 is autotrophic archaea.

### 3.2. Morphology and Physiological Characteristics of Strain DL9LZB001

**Figure 2:**
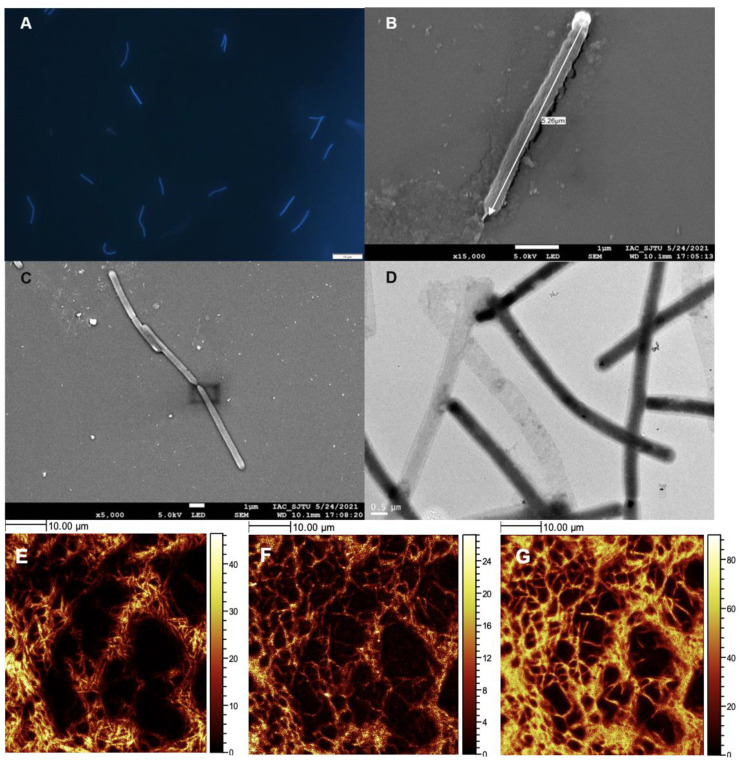
Cells of *Methanothermobacter tengchongensis* DL9LZB001 under a microscope and in ToF-SIMS images. (**A**) Under a fluorescence microscope with 405 nm ultraviolet light as the light source. (**B**,**C**) Under a scanning electron microscope. The length of DL9LZB001 in these images is 5.26 μm, and the diameter is approximately 0.4 μm. (**D**) DL9LZB001 cells under a transmission electron microscope. (**E**–**G**) ToF-SIMS ion images of dried cultured cell aggregates showing their morphology and components. The lighter parts in the pictures are signals of (**E**) C_2_SO_2_^+^, (**F**) a combination of PO_3_^−^, PO_2_^−^, and PO^−^, and (**G**) CN^−^. The darker parts are sections without signal (without cells). The scale bar on the right displays the strength of the signal.

Strain DL9LZB001 was directly observed under a fluorescence microscope (for details, see Section 2.3 and Figure 2A). The DL9LZB001 cells were straight slender rods 2.80–6.43 μm in length (average length = 4.72 ± 1.01 μm, *n* = 9), with an average diameter of 0.436 ± 0.052 μm (*n* = 9). The cells often occurred in pairs, similar to *Methanothermobacter marburgensis* Marburg (Figure 2A–C). *M. marburgensis* Marburg is a pure-cultured strain isolated from the sewage sludge of an artificial digestor (Table 1) and has the closest ANI match to strain DL9LZB001 [48]. We did not observe a flagellum (Figure 2B–D), indicating that the strain DL9LZB001 is nonmotile, similar to most strains in the genus *Methanothermobacter* [17]. We performed ToF-SIMS analysis and 3D surface reconstruction. C_2_SO^+^, PO_2_^−^, and CN^−^ fragments (Figure 2E–G) were employed to represent the cell components.

**Figure 3:**
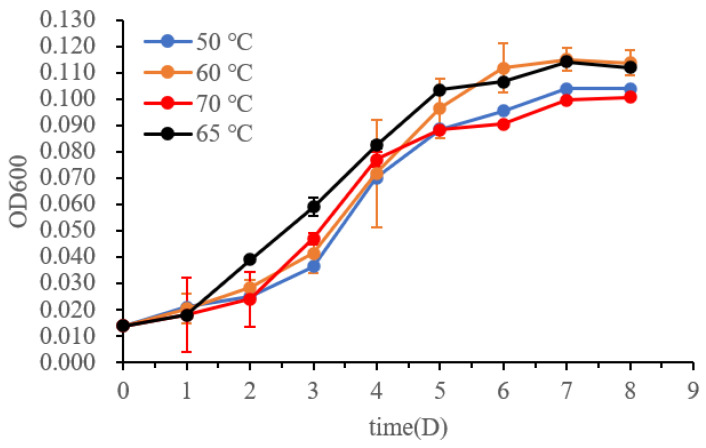
Growth curves of *M. tengchongensis* DL9LZB001 at different temperatures.

The temperature range for growth was tested by measuring the turbidity (OD_600_) of the pure cultures at temperatures of 40, 50, 60, 65, 70, and 80 °C (Figure 3, *n* = 3). No growth was observed at 40 °C or 80 °C. The culture maintained at 65 °C grew better than those at 50, 60, and 70 °C during the exponential growth phase. Therefore, the optimum growth temperature for *M. tengchongensis* DL9LZB001 is approximately 65 °C, close to that of *M. marburgensis* Marburg (Table 1) and the environmental temperature of the sample location. The pH and salinity (NaCl%, *w*/*v*) ranges for growth at 65 °C for the strain DL9LZB001 are pH 6–8 and ≤1%, respectively (Table 1, *n* = 3); both values are less than those for *M. marburgensis* Marburg (Table 1).

### 3.3. General Genome Characteristics of the Strain DL9LZB001

**Figure 4:**
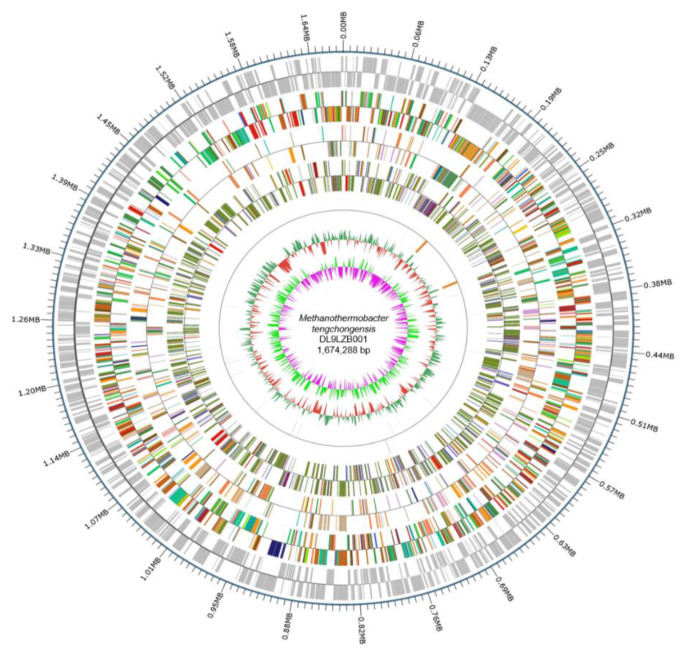
The entire genome map of *M. tengchongensis* DL9LZB001. The outermost circle is the position of the genome sequence. From the outside to the inside: coding genes, gene function annotation results, ncRNA, genome GC contents, and genome GC skew values. We calculated the GC contents and GC skew values based on window bp (chromosome length/1000) and step bp (chromosome length/1000). The red inward part indicates that the GC content in that area is lower than the average of the entire genome, while the green outward part shows higher GC content. The heights of the peaks indicate the difference in value between the GC content of that area and the average GC content. The algorithm of the skew value is (G – C)/(G + C), and the pink inward part indicates that the content of G in that area is lower than that of C, while for the light green outward part the reverse applies. The heights of the peaks indicate the value difference.

Sequencing of the strain DL9LZB001 genome on the PacBio platforms generated 108,026 reads, accounting for 1,072,592,174 bases of the total sequence information. The final assembly and correction using Illumina PE150 produced a closed circular chromosome of DL9LZB001 with a size of 1,674,288 bases and a GC content of 48.39% (Figure 4). Gene prediction using GeneMarkS revealed 1802 protein-coding genes, accounting for 1,522,086 bases (90.91% of the chromosome size), and the number of tRNA genes was found to be 36 (Table 2). Using IslandPath-DIOMB we identified six genomic islands in the chromosome, accounting for 69,449 bases (4.15% of the chromosome size). No predicted prophage gene was identified in the chromosome; however, four CRISPR sequences were predicted by the CRISPRdigger software (Figure 4).

**Figure 5:**
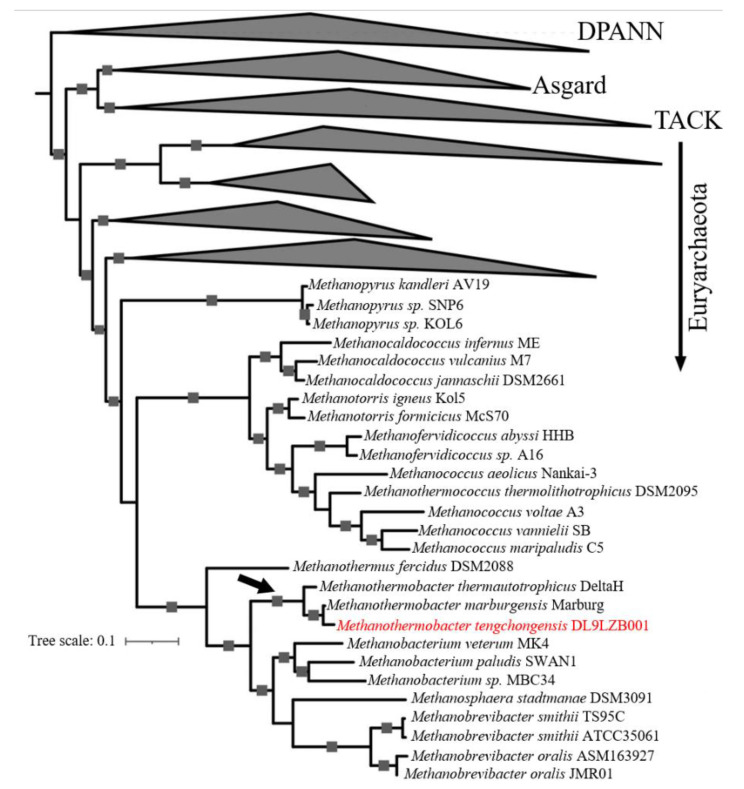
Phylogenomic affiliation of *M. tengchongensis* DL9LZB001. We constructed the phylogenomic tree based on 37 marker-protein sequences, with 233 representative archaea genomes from four superphyla: (from top to bottom) DPANN, Asgard, TACK, and Euryarchaeota [10]. The tree is rooted in the superphylum DPANN. The position of the genus *Methanothermobacter* is labeled with a black arrow, and DL9LZB001 is highlighted in red. Bootstrap values larger than 80 are marked as squares on the tree branch.

To determine the phylogenetic position of the strain DL9LZB001, a phylogenetic tree was constructed based on 37 marker protein sequences with 233 representative archaea genomes from four superphyla: DPANN, Asgard, TACK, and Euryarchaeota (Supplementary Table S2) [10]. The constructed phylogenetic tree is rooted in the superphylum DPANN and displays only the phylogeny of the Class I methanogens. Among the Class I methanogens, the class *Methanobacteria* is clustered with the classes *Methanopyri* and *Methanococci* of the phylum Euryarchaeota (Figure 5). The strain DL9LZB001 is clustered with *M. marburgensis* Marburg and *M. thermautotrophicus* DeltaH within the genus *Methanothermobacter* (Figure 5). Genome comparison results indicate that the strain most closely related to DL9LZB001 is *M. marburgensis* Marburg; the ANI between *M. marburgensis* Marburg and strain DL9LZB001 is 94.13% with an AAI of 94.87% (Supplementary Tables S4 and S5), indicating that the strain DL9LZB001 may represent a novel species, named *Methanothermobacter tengchongensis* DL9LZB001.

**Figure 6:**
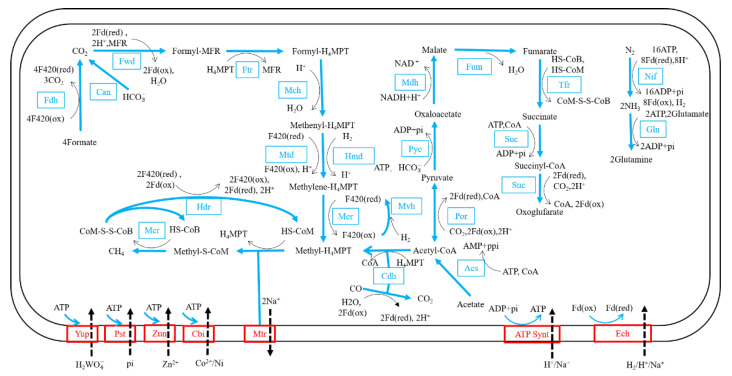
Reconstructed metabolic pathways of *M. tengchongensis* DL9LZB001. We reconstructed the main pathways based on the KEGG annotation. All enzymes and reaction directions are marked in blue, except for enzymes belonging to transporters which are marked in red with dashed lines to show transporting directions.

The metabolic pathways of the strain DL9LZB001 were constructed based on the Kyoto Encyclopedia of Genes and Genomes (KEGG) (Figure 6). DL9LZB001 uses CO_2_ or bicarbonate as the electron acceptor and H_2_ as the electron donor for the hydrogenotrophic methanogenesis pathway, which comprises seven independent enzymic reaction steps (Figure 6). Briefly, the CO_2_ is first reduced to formyl-methanofuran (formyl-MFR) by formylmethanofuran dehydrogenase (Fwd). The formyl group is then transferred to tetrahydromethanopterin (H_4_MPT). The formyl-H_4_MPT is dehydrated to methenyl-H_4_MPT, reduced to methylene-H_4_MPT, then reduced to methyl-H_4_MPT (Figure 6). Then, the methyl group is transferred to the coenzyme M (HS-CoM) by methyl-H_4_MPT methyltransferase (Mtr), a sodium ion transporter. The final and rate-limiting step of the methanogenesis pathway is the reduction of the methyl coenzyme M (methyl-S-CoM) by methyl-coenzyme M reductase (Mcr), with the coenzyme B (HS-CoB) as the electron donor. For recycling HS-CoM and HS-CoB, coenzyme M and coenzyme B heterodisulfide (CoM-S-S-CoB) are reduced by the heterodisulfide reductase (Hdr). During this process, three coenzyme F420 (reduced) molecules and one H_2_ molecule or four coenzyme F420 (reduced) molecules are consumed in total to generate one methane molecule (Figure 6). The reduction of coenzyme F420 (oxidized) is catalyzed by methyl-viologen-reducing hydrogenase (Mvh), with H_2_ as the electron donor. Therefore, four H_2_ molecules are consumed to generate one methane molecule. DL9LZB001 also contains formate dehydrogenase (Fdh), which can catalyze four formate molecules to four CO_2_ molecules and produce four coenzyme F420 (reduced) molecules for the subsequent generation of one methane molecule.

Our annotation results indicate that DL9LZB001 may also use acetate for methanogenesis (Figure 6). The first step is acetate activation by acetyl-CoA synthetase (Acs) with ATP as the energy donor. The acetyl-CoA is then cleaved into methyl and carbonyl groups by acetyl-CoA decarbonylase (Cdh). However, we failed to detect methane production via pure culturing under 100% N_2_ of DL9LZB001 with 10 mM acetate and 5 g·L^−1^ bicarbonate as the only carbon source (65 °C, pH7, 0.2% NaCl). The known acetoclastic methanogens primarily belong to the order *Methanosarcinales* and the genus *Methanothrix* [52,53]; however, no acetate-utilizing ability has been reported in the genus *Methanothermobacter*. Therefore, the actual functions of Acs and Cdh require further research. The strain DL9LZB001 uses an incomplete tricarboxylic acid cycle for carbon fixation and may have the potential to transform nitrogen to ammonia (Figure 6). No intact sulfur pathway was discovered in this strain. We found only that it may have the potential to oxidize thiosulfate to sulfite using the enzyme thiosulfate sulfurtransferase, or to sulfate using S-sulfosulfanyl-L-cysteine sulfohydrolase. It also includes several ABC-type transporters for the uptake of tungstate, phosphate, zinc, cobalt, and nickel, with ATP as the energy driver (Figure 6).

### 3.4. The Evolutionary History of the Class Methanobacteria

**Figure 7:**
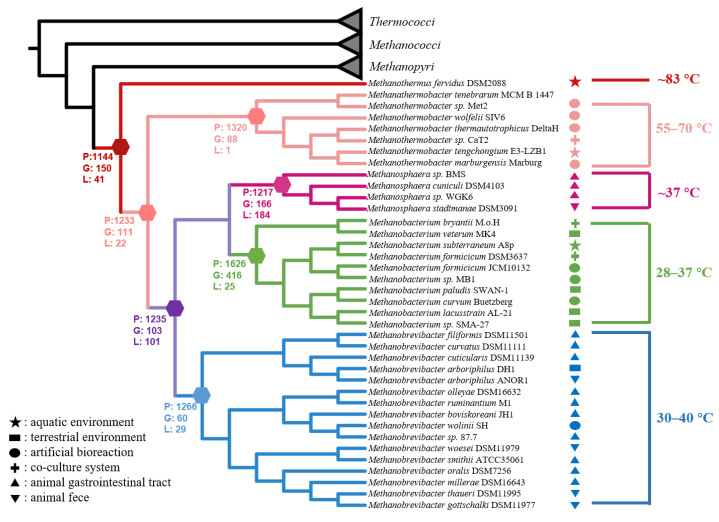
Phylogenomic tree of the class *Methanobacteria*, based on 37 marker protein sequences with the completely sequenced genomes of isolated strains from *Thermococci*, *Methanococci*, *Mathanopyri*, and *Methanobacteria*. We rooted the tree in the class *Thermococci1* and labeled the optimum growth temperature ranges with corresponding colors as the genus within the class *Methanobacteria*. The number of present genes (P), gained genes (G), and lost genes (L) on the speculated common ancestor nodes were also labeled. The genus *Methanothermus* (83 °C) is the deepest clade, followed by the genus *Methanothermobacter* (55–70 °C). Other clades are the genera *Methanobrevibacter* (30–40 °C), *Methanosphaera* (37 °C), and *Methanobacterium* (28–37 °C). We also labeled the in situ environments of strains with different geometries. The star denotes that the strains were isolated from natural aquatic environments, including hot springs or groundwater. The rectangle denotes that the strains were isolated from natural terrestrial environments, including permafrost, peat soil, and wet wood. The ellipse indicates that the strains were isolated from artificial bioreactions, including digesters and fermenters. The plus sign indicates that the strains were isolated from co-culture systems. The regular triangle indicates that the strains were isolated from gastrointestinal tracts of animals including mammals and termites. The del operator indicates that the strains were isolated from animal feces including those from mammals and geese.

For evolutionary analyses, we collected from the GTDB database all completely sequenced genomes of the isolated strains from the classes *Thermococci*, *Methanobacteria*, *Methanococci*, and *Methanopyri* (https://gtdb.ecogenomic.org/ (accessed on 26 February 2022), Supplementary Table S1), including the strain DL9LZB001. A phylogenetic tree was constructed, rooted in the class *Thermococci* (Figure 7). We also added to the phylogenetic tree the environmental information corresponding to each isolated strain (Figure 7). Most of the published pure cultured and completely sequenced strains of the genus *Methanothermobacter* were isolated from artificial environments (Figure 7), whereas DL9LZB001 was isolated from a natural hot spring (Figure 1). Adding this newly isolated strain from a natural environment to construct the evolutionary history of the class *Methanobacteria* may increase the habitat diversity on the ancestral node of the genus *Methanothermobacter*. All the COG of these genomes were identified using the Orthofinder software and were subsequently annotated. The gene sets of ancestor nodes were predicted by a posterior probability algorithm in the Count software, and the gene set of the common ancestor of the class *Methanobacteria* was constructed.

Based on the KEGG annotation, the common ancestor of the class *Methanobacteria* possesses hydrogenotrophic pathways, and it can use formate for methanogenesis (Supplementary Table S3). However, none of the known nitrogen and sulfur metabolic pathways are complete, indicating that it may have alternative pathways for nitrogen and sulfur assimilation. The *Methanobacteria* ancestor also codes for most subunits of phosphate, tungstate, molybdate, zinc, cobalt, nickel, and biotin ABC-type transporters. Previous phylogenomic analysis suggests that the Class I methanogens including the class *Methanobacteria* may have originated before the Great Oxygenation Event (GOE), which is consistent with their strictly anaerobic features [54]. There have also been studies focused on the evolution of the genus *Methanosphaera* within the class *Methanobacteria* that confirm its monophyletic origin [55]. Nonetheless, to our knowledge, no research has systemically focused on the evolutionary history of the class *Methanobacteria*.

Based on the phylogenetic results and environmental information, we found a clear trend indicating that the optimum growth temperatures for different genera decreased gradually during the evolution of the class *Methanobacteria*. At the deep-branching position of the class *Methanobacteria*, the first diverging clade was the genus *Methanothermus* (83 °C), followed by the genus *Methanothermobacter* (55–70 °C), with other members of the class being the mesophilic genera (28–40 °C) including *Methanobrevibacter*, *Methanosphaera*, and *Methanobacterium* (Figure 7). Therefore, we hypothesize that the loss of genes related to thermal adaptation by the ancestor nodes led to mesophilic adaptation.

Compared with the deep-branching genus *Methanothermus*, other nodes lost genes related to the reverse gyrase gene (*rgy*) (Supplementary Table S3). By consuming ATP, Rgy can modify the topological state of DNA by introducing positive supercoils, which are very important for the hyperthermal adaptation of DNA and may also be involved in unwinding DNA strands under a positive supercoil state to allow subsequent transcription or replication [56]. We propose that the loss of *rgy* might be one of the factors that altered the living conditions of the common ancestor of the genus *Methanothermobacter* and other mesophilic methanogens, resulting in a shift in their temperature adaptation ability from hyperthermal (83 °C) to thermal or ambient adaptation (28–70 °C). By comparing the common ancestor of the thermal methanogens (50–70 °C) and mesophilic methanogens (28–40 °C), we found that the latter lost the genes coding for enzymes that may be related to thermal adaptation—such as the integral membrane protein that may help to stabilize the membrane under thermal conditions, and the enzyme that repairs DNA, prevents DNA lesions, or facilitates DNA transcription (Supplementary Table S3)—which may have helped the methanogens to adapt to thermal environments.

The three mesophilic genera *Methanobrevibacter* (30–40 °C), *Methanosphaera* (37 °C), and *Methanobacterium* (28–37 °C) cluster together and share a mesophilic common ancestor (Figure 7). However, the methanogenesis pathway of the genus *Methanosphaera*, which gains energy by reducing methanol to methane only when H_2_ is the electron donor, is the most specific in the entire class *Methanobacteria*. Our results show that the common ancestor of the genus *Methanosphaera* lost genes coding for the complex that catalyzes the reversible cleavage of acetyl-CoA and enzymes related to the hydrogenotrophic methanogenesis pathway, i.e., the sulfur-carrier protein required for the activity of Fdh, the subunit E of the Fwd, coenzyme F420-dependent NADP oxidoreductase, and even part of the Mtr complex related to energy conservation (Figure 6, Supplementary Table S3). However, it gained genes encoding for enzymes, such as methanol cobalamin methyltransferase, corrinoid iron-sulfur protein (which transfers methyl in the Wood-Ljungdahl pathway), and the subunit A of methanol-coenzyme M methyltransferase (MtaA). Mta catalyzes methyl group transfers from methanol to coenzyme M to form methyl coenzyme M [55]. We propose that gaining these genes occasioned the transformation of the genus *Methanosphaera* from hydrogenotrophic metabolism to hydrogen-dependent methanol metabolism.

Interestingly, strains of the genera *Methanosphaera* and *Methanobrevibacter* were all isolated from the gastrointestinal systems of mammals or termites or the feces of mammals or geese, except for *Methanobrevibacter arboriphilus* DH1, which was isolated from wet wood, and *Methanobrevibacter smithii* ATCC35061 from a primary sewage digester (Figure 7) [18,20,57]. However, all the strains of the genus *Methanobacterium* were isolated from natural systems (Figure 7) [19]. Compared with the genus *Methanobacterium*, which inhabits natural environments, the common ancestor of the host-associated genus *Methanobrevibacter* did not experience considerable gene gain or loss (Figure 7, Supplementary Table S3). The potential reason for this may be that both genera continue to depend on H_2_/CO_2_ utilization as the anaerobic intestine environments of the host may be similar to ancient natural anaerobic environments. However, the common ancestor of another host-associated genus, *Methanosphaera*, experienced significant gene gain and gene loss, and obtained energy from the hydrogen-dependent reduction of methanol to methane (Supplementary Table S3). Previous research indicates that the methanol concentration in gastrointestinal systems (10 μM in cockroaches, 23–72 μM in the rumen, and 70 μM in humans) is higher than in natural environments where methanol is generally around or below the micromolar level [58,59,60,61]. Therefore, this specific selection force may have driven the ancestor of the genus *Methanosphaera* to become better adapted to methanol-rich gastrointestinal systems. Therefore, the host-adapted genera *Methanobrevibacter* and *Methanosphaera* may have evolved independently to utilize different substrates in intestinal systems. It should be fascinating to study what produced this massive difference between these two closely related genera, which inhabit similar environments.

## 4. Conclusions

Methanogens are one of the most ancient life forms on Earth and have immensely impacted the global climate since their origin. They exist in an extensive range of all kinds of anaerobic ecosystems and are considered to have originated in geothermal environments. In this study, we isolated and pure cultured one methanogen, temporarily named *Methanothermobacter tengchongensis* DL9LZB001, from a hot spring at Tengchong and completely sequenced its genome. DL9LZB001 utilizes H_2_ and CO_2_ for methanogenesis, with an optimum growth temperature of 65 °C. Genome analysis demonstrates that it has a complete hydrogenotrophic methanogenesis pathway and a potential acetate metabolic pathway. Phylogenetic analysis indicates that the strain DL9LZB001 belongs to the class *Methanobacteria* of the Class I methanogens, and its closest relative is the strain *M. marburgensis* Marburg. Comparative genomic and ancestral analysis of isolated strains among the Class I methanogens indicates that the class *Methanobacteria* had a hyperthermal origin, from which it gradually evolved to be thermophilic and then finally to be mesophilic. The evolutionary history of the class *Methanobacteria* is an odyssey of leaving high-temperature niches, which have the greatest potential to be cradles of life. These ancient strains gradually lost their thermal adaptation and came to occupy niches with different temperatures.

## Figures and Tables

**Table 1:** Descriptive and catabolic features of *M. tengchongensis* DL9LZB001 compared with other members of the genus *Methanothermobacter*.

Characteristics	1 *	2 *	3 *	4 *	5 *	6 *	7 *	8 *
Origin	Gas field	Sewage sludge	Anaerobic sludge	Anaerobic sludge	Anaerobic sludge	Sewage sludge and river sediment	Sewage sludge digester	Hot spring upper water and sediment
Cell size (diameter × length, μm × μm)	0.5 × 3.5–10.5	0.35–0.6 × 3–7	0.40 × 7–20	0.36 × 1.4–6.5	0.42 × 3–6	0.35–0.5 × 2.5	0.3–0.4 × 3–3.5	0.436 × 2.80–6.43
Filaments	–	+	+	+	–	+	+	+
Gram stain results	+	+	+	–	+	+	+	+
Optimum temperature (°C)	70	65–70	55	57	60–65	55–65	65	60–65
pH range for growth	5.8–8.7	6.0–8.8	7.5–8.5	7.0–8.5	6.0–7.5	6.0–8.2	5.0–8.0	6.0–8.0
Max NaCl concentration for growth (*w*/*v*, %)	2	n. d.	3	3	2	1	3.5	1
Growth with formate	–	–	+	–	+	+	–	+
Autotrophic	–	+	+	+	+	+	+	+
Dependent growth factors:								
Acetate	–	–	–	n. d.	–	–	–	–
Yeast extract	+	–	–	n. d.	–	–	–	–
Coenzyme M	n. d.	–	+	+	–	n. d.	–	–
Peptone	+	–	–	n. d.	–	n. d.	–	–

^*^ Strains/species: 1 → *M. tenebrarum* RMAS [22], 2 → *M. thermautotrophicus* DeltaH [48,49], 3 → *M. thermoflexus* IDZ [50], 4 → *M. thermophilus M* [17], 5 → *M. defluvii* ADZ [50], 6 → *M. wolfeii* DSM 2970 [51], 7 → *M. marburgensis* Marburg [48], 8 → *M. tengchongensis* DL9LZB001 (this study). n.d. indicates that there were no related tests. For the symbols see the standard definitions.

**Table 2:** General genome features of *M. tengchongensis* DL9LZB001.

General Features	*M. tengchongensis* DL9LZB001
Size (bp)	1,674,288
GC content (%)	48.39
Protein coding genes	1802
Genomic islands	6
CRISPR ^1^ sequences	4
Genes assigned to COG ^2^ categories	1487
tRNA	36
5S rRNA	3
16S rRNA	2
23S rRNA	2
sRNA	0

^1^ CRISPR: Clustered regularly interspaced short palindromic repeat. ^2^ COG: Clusters of orthologous group.

## Data Availability

Genome sequence can be downloaded from eLMSG (https://www.biosino.org/elmsg/index) with code: https://www.biosino.org/elmsg/record/MSG083741?code=4644.

## Supplementary Materials

The following supporting information can be downloaded at https://www.mdpi.com/article/10.3390/biology11101514/s1, Table S1: environmental information of isolated strains within the class *Methanobacteria*; Table S2: information of 37 marker-protein sequences; Table S3: common ancestor gene annotations within the class *Methanobacteria*; Table S4: AAI from strain DL9LZB001 to the isolated strains within the class *Methanobacteria*; Table S5: ANI from strain DL9LZB001 to the isolated strains within the class *Methanobacteria*.
